# StochPy: A Comprehensive, User-Friendly Tool for Simulating Stochastic Biological Processes

**DOI:** 10.1371/journal.pone.0079345

**Published:** 2013-11-18

**Authors:** Timo R. Maarleveld, Brett G. Olivier, Frank J. Bruggeman

**Affiliations:** 1 Systems Bioinformatics, Amsterdam Institute for Molecules Medicines and Systems, VU University Amsterdam, Amsterdam, The Netherlands; 2 Life Sciences, Centrum Wiskunde & Informatica, Amsterdam, The Netherlands; 3 BioSolar Cells, Wageningen, The Netherlands; 4 Kluyver Centre for Genomics of Industrial Fermentation, Delft, The Netherlands; Université de Nantes, France

## Abstract

Single-cell and single-molecule measurements indicate the importance of stochastic phenomena in cell biology. Stochasticity creates spontaneous differences in the copy numbers of key macromolecules and the timing of reaction events between genetically-identical cells. Mathematical models are indispensable for the study of phenotypic stochasticity in cellular decision-making and cell survival. There is a demand for versatile, stochastic modeling environments with extensive, preprogrammed statistics functions and plotting capabilities that hide the mathematics from the novice users and offers low-level programming access to the experienced user. Here we present StochPy (*Stoch*astic modeling in *Py*thon), which is a flexible software tool for stochastic simulation in cell biology. It provides various stochastic simulation algorithms, SBML support, analyses of the probability distributions of molecule copy numbers and event waiting times, analyses of stochastic time series, and a range of additional statistical functions and plotting facilities for stochastic simulations. We illustrate the functionality of StochPy with stochastic models of gene expression, cell division, and single-molecule enzyme kinetics. StochPy has been successfully tested against the SBML stochastic test suite, passing all tests. StochPy is a comprehensive software package for stochastic simulation of the molecular control networks of living cells. It allows novice and experienced users to study stochastic phenomena in cell biology. The integration with other Python software makes StochPy both a user-friendly and easily extendible simulation tool.

## Introduction

Experiments at the level of single cells indicate large cell-to-cell variability in copy numbers of molecules [1]. Inevitably, this molecular noise originates from stochastic fluctuations and has a large impact on cellular dynamics [2]. Traditional deterministic chemical kinetics fail to capture the dynamics of these systems. Stochastic systems are typically mathematically described by the master equation [3], which rarely has a closed form solution and therefore numerical simulation is a necessity. Stochastic simulation algorithms (SSAs) generate time trajectories that are in agreement with the master equation. Many SSAs have been developed [4], however, in order to effectively use them in cell biology a flexible simulation environment is required. This should include, for instance, easy access to functionality for statistical analysis, plotting and interpretation of the raw simulation results, all the while shielding the modeler from the underlying mathematics.

Stochastic simulation software is used in a variety of modeling methodologies e.g. ordinary differential equations [5]–[11] and Petri nets [12], each tool having its own unique strengths and weaknesses. However, the recent advances in the experimental investigation of single-cells has increased the interest in the analysis of the statistics of event waiting times [13]–[16]. Most simulators cannot calculate these event waiting times because they do not return the raw stochastic simulation output (hereafter explicit output). In addition, stochastic modeling is not as straightforward as solving deterministic systems and there is a demand for a versatile stochastic modeling environment that is easy to use and extend. Altogether, this motivated us to develop a flexible and interactive open-source stochastic simulator StochPy: *Stoch*astic modeling in *Py*thon.

StochPy provides various SSAs for the simulation of stochastic dynamics and supports model definition in either plain text or the Systems Biology Markup Language (SBML) [17]. In addition it includes statistical functions for the numerical analysis of stochastic simulations as well as plotting facilities for the visualization of amongst other features time-correlations, propensities, and event waiting times.

## Results and Discussion

### Software Implementation

The StochPy software has been designed around three core principles. *Accessibility*, it should not be limited to a specific operating system or user environment. *Functionality*, it should implement a variety of SSAs, allow for the intuitive description of models (i.e. reactions and species), and provide high-level, user-friendly access tailored for interactive use. *Flexibility*, it should support high-level statistical and plotting functions for interrogating both model and data as well as provide programmatic access to low-level functions and data structures.

To satisfy these principles StochPy has been developed as a console application using the Python language, taking advantage of its pure object-oriented nature, portability, extensive standard library, and ability to seamlessly glue together scientific libraries written in compiled languages. For instance, Matplotlib [18] is integrated for plotting, providing publication-quality image generation. An object-oriented design allows for the simultaneous analysis of multiple instances of a model, using state-of-the-art stochastic simulation capabilities either interactively or via user-defined scripts. Note that, because of the high-level functionalities, knowledge of the Python programming language is not required although, any scripting knowledge will enhances the modeling experience.

Combining these functionalities with those provided by the many available Python scientific libraries allows for easy extension of StochPy as well as its use as a library in other simulation software. The StochPy software has already been incorporated as a plug-in library for the systems biology simulator software PySCeS [19]. This provides a single interactive environment where model properties (e.g. parameters and species amounts) can be set interactively and simulated in both a stochastic and deterministic manner.

The following SSAs are implemented in StochPy: The direct, first reaction, next reaction, and optimized tau-leaping methods [4], [20], [21]. Whilst the direct method is selected by default, the next reaction, and tau-leaping methods can be used to boost performance for models that are either sparse or have many fast reactions and/or large molecule numbers, respectively. These implementations successfully passed all tests from the SBML stochastic test suite [22], the results of which are given in Dataset S1.

Model definition is by way of the human readable/writable PySCeS model description language (MDL) [19]. A simple and intuitive approach to creating and editing models understandable to experimentalists and theoreticians. Moreover, libSBML [23] is used for importing stochastic models written in SBML format which are then subsequently translated into PySCeS MDL. This requires maintaining one MDL that supports features which in principle can be encoded in any SBML instance. Currently, StochPy implements SBML Level 2 version 4 import and export functionalities. SBML Level 3 and package support is now being investigated.

The StochPy software provides interfaces to other widely used simulation tools: CAIN and StochKit2. Through these integrated interfaces, modelers are provided with a choice of SSA implementations that differ in speed and simulation output. For example, using StochKit2 via StochPy allows simulating models defined in SBML up to L2V4 or PySCeS MDL by StochKit2’s fast solvers. The output can then directly by analyzed in StochPy, without the need to install any additional software (by default StochKit2 uses MATLAB to provide this functionality).

### Performing StochPy Simulations

A typical StochPy modeling session consists of first creating a StochPy model object from a (default) input model. Alternatively, different user-defined models (in SBML or PySCeS MDL) can be loaded into the model object. Once a model is loaded various simulation parameters can be set, e.g. the number of simulation steps and the number of simulation trajectories. Subsequently, kinetic parameter values and species amounts can be modified interactively, and simulations can be performed by calling the available analysis methods for model objects. As model objects are fully encapsulated, multiple models can be instantiated from the same (or different) input files at the same time. An example of a short modeling session within Python can be,

>>> import stochpy

>>> smod = stochpy.SSA()

>>> smod.Model(‘dsmts-001-01.xml’)

>>> smod.DoStochSim(end = 1000,mode = ‘steps’)

>>> smod.PlotSpeciesTimeSeries().

Here, we initiate the model object smod for the default input model, load a different model depicted in SBML into the model object smod, generate one time trajectory of the master equation (1000 steps), and plot the corresponding (discrete) species time series data.

In the following sections we discuss the potential uses of explicit output in systems biology, highlight StochPy’s capabilities by modeling different biological systems, and benchmark StochPy against other widely used stochastic tools. All simulations were done with StochPy’s implementation of the direct method. Only a single command, a high-level function such as PlotSpeciesTimeSeries(), is necessary to create most of the shown (sub)-figures. Annotated scripts and input files used to generate modeling results are available as Scripts S1. More information on installing and using StochPy can be found in the *StochPy User’s Guide* which together with additional example sessions is available online at http://stochpy.sf.net.

### The Potential Uses of Explicit Output in Systems Biology

StochPy returns explicit output rather than discretized output (hereafter fixed-interval output). With fixed-interval output we mean that the state of the system, i.e. the copy numbers of all molecules, are reported for fixed-time intervals and not at the times in the system when single reaction events occur. Mathematically speaking, molecular reaction systems are modeled by continuous-time discrete state Markov chains and fixed-interval data storage approaches simulate these systems with continuous time but store the output with fixed-time intervals. Returning fixed-interval output likely derives from stochastic modeling practices in mathematical statistics. In systems biology, the requirements for stochastic simulation are often different. Access to exact simulation times allows for the straightforward calculation of event waiting times, species and propensity distributions, and correlation times. As these quantities are in principle observable in single-cell experiments, they should be calculable with simulation software.

**Figure 1:**
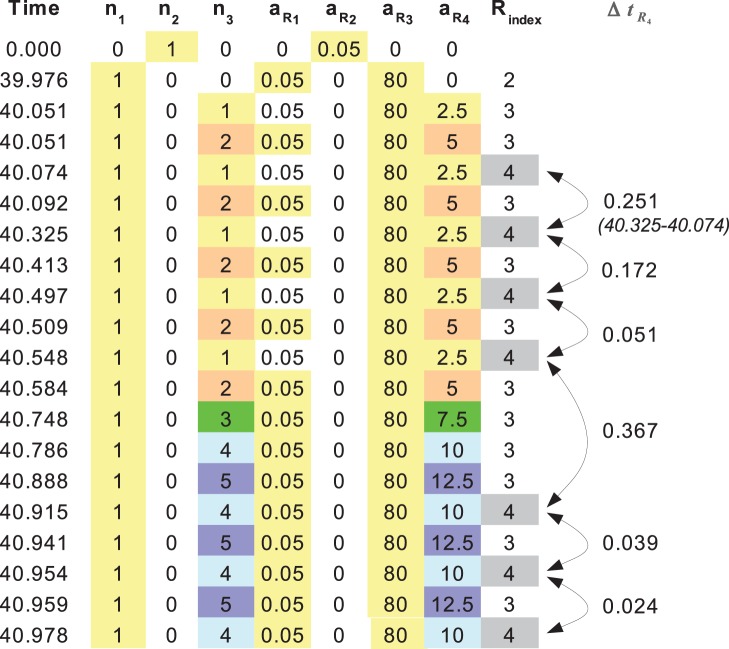
StochPy simulation output. An example of explicit simulation output of StochPy is shown in a table. It reports the number of molecules of each molecular species and the reaction propensities at each time point when a reaction occurs. The time differences between consecutive rows indicate waiting times between reaction events. In the last column, the waiting times for reaction 4, 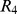, are given and they correspond to the time period between consecutive instances of activity of reaction 4.

Figure 1 illustrates typical output of a StochPy stochastic simulation: It gives 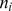, which denotes the number of molecules of molecular species 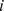 and the propensities (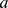) at time points when reaction events occurred. This simulation output can be analyzed using pre-defined statistical functions to plot time series, or calculate distributions, moments, and time-correlations. StochPy returns explicit output and therefore waiting times for particular reactions events or system delays can be calculated. Note that the time between consecutive activities of reactions is called an event waiting time. An example of these event waiting times is shown in Figure 1 where the times between consecutive “firings” of reaction 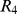 are shown.

**Figure 2:**
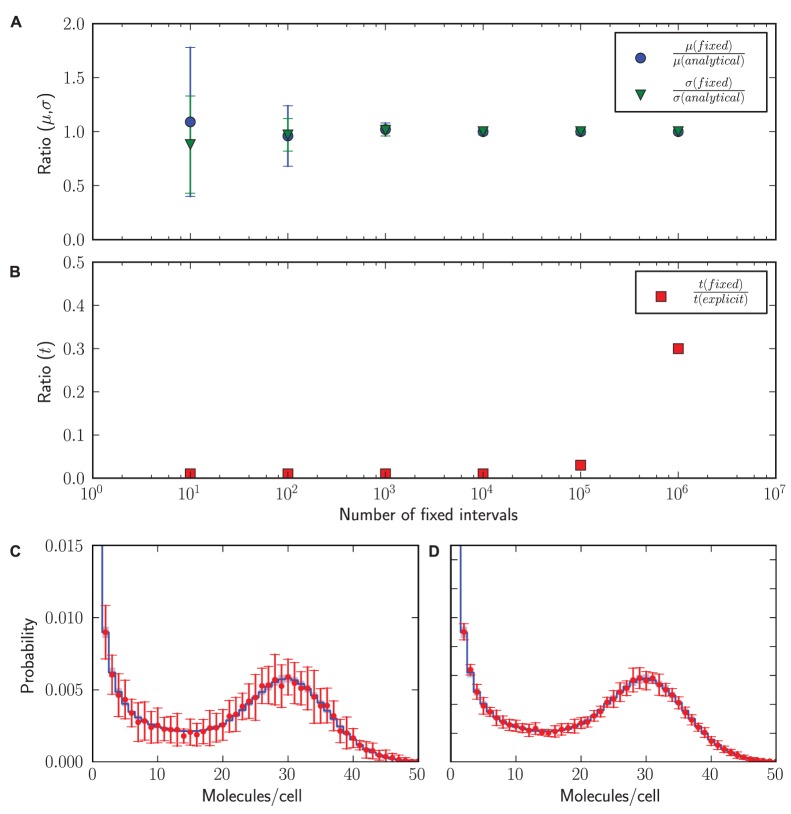
Fixed interval versus explicit simulation output. Hundred stochastic simulations until t = 60.000 min (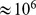 time steps) were done with 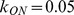 min^−1^, 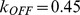 min^−1^, 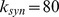 min^−1^, and 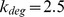 min^−1^. (A) Accuracy of mean and standard deviation estimates as function of the number of fixed intervals. (B) Simulation time with fixed-interval output increases with the number of fixed intervals. Fixed-interval simulations were done with the StochPy interface to StochKit2 and include the time to calculate the associated probability distributions. (C) The stationary mRNA distribution for 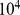 fixed intervals (red error bars, 1.96 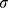) vs. explicit output (blue 95% confidence interval). Note that 1.96 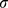 corresponds to a 95% confidence interval. (D) The stationary mRNA distribution for 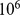 fixed intervals (red error bars, 1.96 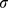) vs. explicit output (blue 95% confidence interval).

In Figure 2 we highlight several differences between fixed-interval and exact output. This example concerns the model specified in Section 3 of Information S1. We ran a stochastic simulation at a stationary state of the model with StochPy to obtain 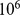 reaction events and obtained all the stochastic dynamics in terms of the exact output. Next, we ran StochKit2 until the same model end time, as we obtained with StochPy, and varied the fixed-interval size at which the state of the stochastic system is stored in the report file of StochKit2. In Figure 2A we plot the estimate of mean (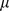) and the standard deviation (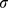), obtained from 100 simulations, as function of the number of fixed intervals. This plot indicates that for this system 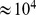 intervals are enough to estimate 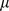 and 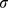 with good accuracy. Figure 2B shows the ratio of the calculation time of StochKit2 and StochPy and indicates that StochKit2 is indeed a lot faster as it only required to write 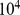 events to file as compared to StochPy, which stored 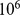 events. StochKit2’s C++ implementation makes the simulation about a factor of 10 faster than StochPy when they store an equal amount of reaction events. Calculating the associated stationary probability distribution required additional time which reduced the speed difference to a factor of 3.

A complication with fixed-interval storage is that the user does not know beforehand what the relevant fixed-interval size and number should be and, for instance, data bootstrapping should be applied to assess the accuracy of the calculation. For instance, 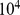 fixed-intervals are not enough to determine the stationary probability distribution with great accuracy, as shown in Figure 2C (the error bars denote the variation in the distributions between the 10 simulations done at each fixed-interval number). Using more fixed-intervals results in determining the stationary probability distribution with a better accuracy. However, Figure 2D shows that a better accuracy was obtained using 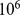 explicit reaction events than with 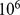 fixed-intervals. Therefore, for this particular example, more fixed-intervals than actual reactions events are necessary to obtain a stationary probability distribution with a similar accuracy.

This means that the speed of a fixed-interval algorithm is not set by the end time and the programming language, as is the case for an exact output approach, but also by the chosen number of fixed intervals. Note that deciding the right number of fixed intervals can only be done by trail and error. Generally, more than 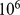 events should be a good starting value, provided that the time-scale separation between reactions within the network is limited.

### Case Study 1: Molecule Synthesis and Degradation

In this section, we modeled the immigration-death model (Information S1 Section 2) which consists of two reactions: A zero-order synthesis reaction of mRNA with rate constant 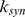 and a first-order mRNA degradation reaction with rate constant 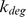. The mRNA synthesis rate, 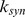, was 10 min^−1^ and the degradation rate constant 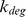 was set to 0.2 min^−1^.

**Figure 3:**
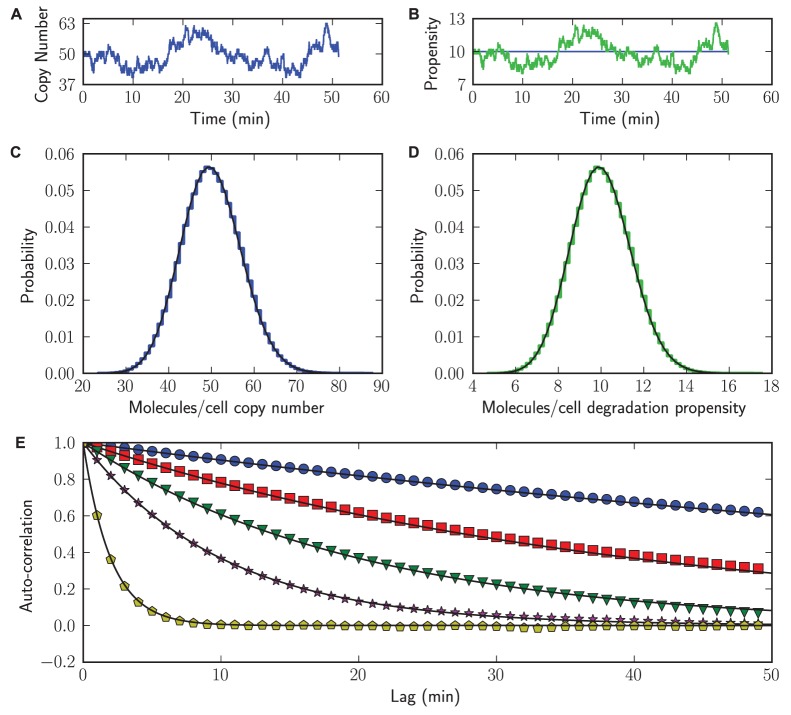
Propensities and auto-correlations. Illustration of several plotting options in StochPy. Colored lines represent StochPy output and black the analytical solutions. (A) species time-series data. (B) propensities time-series data. (C) species distribution. (D) propensities distribution. (E) auto-correlation for different 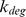 values (0.01, 0.025, 0.05, 0.1, 0.5).

Figure 3A illustrates that the number of mRNA molecules per cell fluctuate around their steady-state copy number of 50 molecules/cell. High mRNA copy numbers correspond to high mRNA degradation propensities (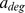; a measure for the reaction rate) and vice versa as is shown in Figure 3B. In contrast, the mRNA synthesis propensity, 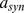, is constant through time.

Distributions can give us more insight into the size of fluctuations. Figure 3C indicates that the mRNA copy numbers are Poisson distributed with a mean of 50 (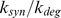). The distribution of 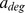 follows a Poisson distribution whose x-axis is distorted (Figure 3D). We also performed an auto-correlation analysis of the mRNA time series (Figure 3E); the auto-correlation time decays exponentially as 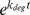 in agreement with theory. For different parameter settings, StochPy simulations match the analytical solution.

### Case Study 2: Stochastic mRNA Synthesis by a Switching Gene

In this section, we consider a model of mRNA synthesis by a gene that switches spontaneously between an inactive (OFF) state and an active (ON) state (Information S1 Section 3). The synthesis of mRNA occurs only during the ON state whilst mRNA degradation occurs continuously. Depending on the choice of kinetic parameters, this system can display transcription bursts that can cause significant cell-to-cell variability in mRNA expression levels [15], [24].

**Figure 4:**
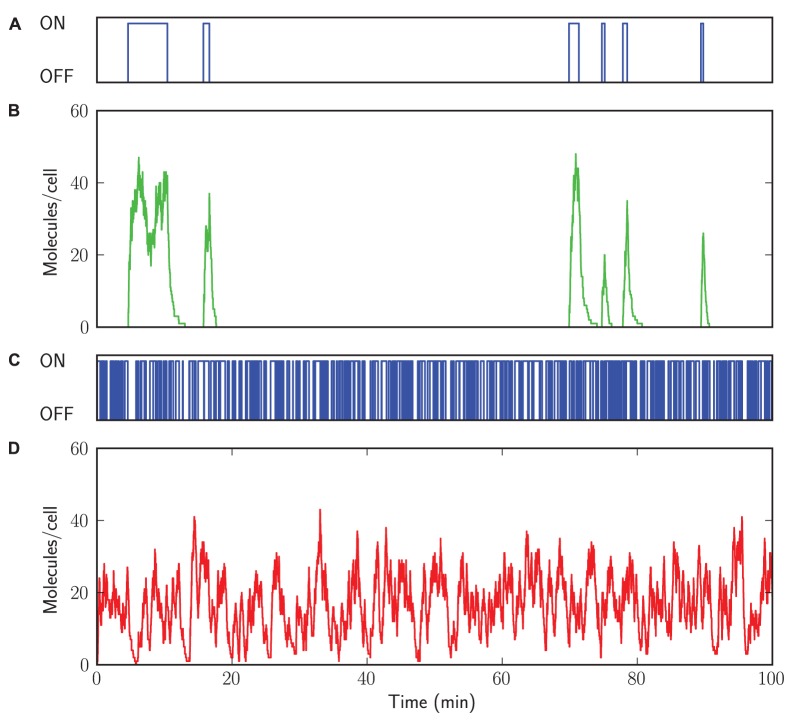
Time series of bursty and non-bursty transcription. StochPy plots of simulating stochastic gene expression. (A) long lifetimes of both the ON and OFF state. (B) bursty transcription. (C) short lifetimes of both the ON and OFF state. (D) non-bursty transcription.

**Figure 5:**
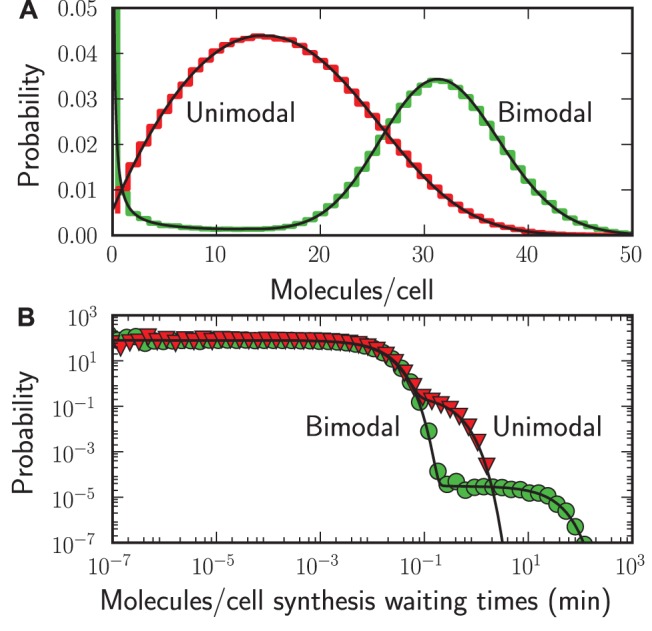
mRNA copy number and event waiting times distributions. StochPy plots of simulating stochastic gene expression with StochPy simulations (step, markers, colored) and analytical solutions (solid, black). (A) probability distribution of the mRNA copy numbers. (B) probability distribution of the mRNA synthesis event waiting times.

In Figures 4 and 5 we compare two kinetic parameter settings leading to a bursty and a non-bursty mode of transcription. Long lifetimes of both the ON and OFF state cause bursty transcription (Figure 4A–B), while short lifetimes of both the ON and OFF state cause non-bursty transcription (Figure 4C–D). These transcription bursts lead to bimodal mRNA copy number distributions (Figure 5A) and two time scales in the distribution of event waiting times of mRNA synthesis times (Figure 5B). These event waiting times depend on both the production during a ON state and the periods of inactivity [15]. These results are in agreement with the analytical solutions (the black solid lines), which are further discussed in Information S1 Section 3.

### Case Study 3: Spontaneous Fluctuations in Single-molecule Enzyme Activity

Next, we consider a completely different model that describes single-molecule enzymology (Information S1 Section 4). A second-order reaction converts an enzyme 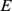 and a substrate 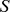 into an enzyme-substrate complex 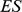. Subsequently, with two different first-order reactions this enzyme-substrate complex can fall apart and return the enzyme and substrate or give rise to the enzyme and the product 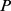.

**Figure 6:**
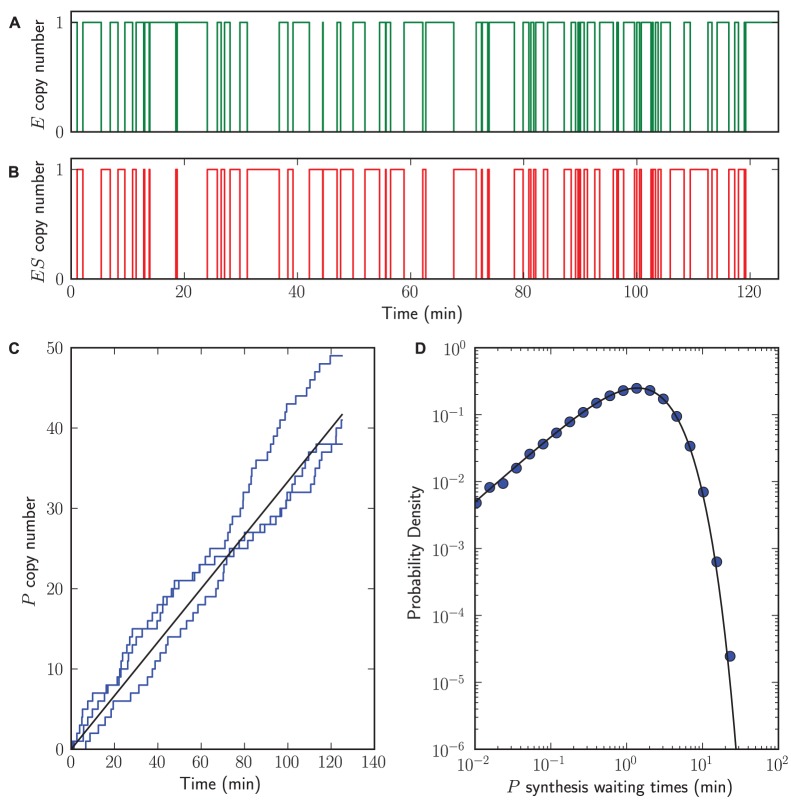
Single-molecule enzymology. StochPy plots for single-molecule enzyme activity simulations with StochPy simulations (step, markers, blue) and analytical solutions (solid, black) (A–B) time-series data of 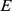 and 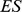, 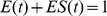. (C) three time trajectories that fluctuate around the analytical solution. This analytical solution corresponds to the mean rate of formation, which for stochastic simulations can be obtained by taking the average of many generated time trajectories. (D) event waiting times of product 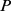 formation peak.

The simulation results are shown in Figure 6. Because we consider a single enzyme molecule, the number of enzyme molecules 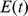 is either 1 or 0 and the same applies to 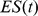 (Figures 6A–B). In Section 4 of Information S1 we show that we can derive the Michaelis-Menten relationship from this model. As a consequence, the rate of formation of product 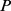 depends on 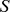, 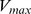, and 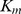. In Figure 6C we visualize both stochastic simulations and the analytical result of product 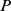 formation as a function of time. Finally, in Section 4 of Information S1 we also show that the waiting times distribution of product 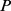 formation is analytically solvable. The waiting times determined with StochPy are in agreement with this analytical solution (Figure 6D).

### Case Study 4: Modeling Cell Division Explicitly and Implicitly

To demonstrate the flexibility of StochPy we briefly illustrate how simple it is to extend a stochastic model of a gene expression network with explicit cell division events, even though this is not a standard functionality of StochPy. In this model, protein synthesis occurs from mRNA and mRNA synthesis depends on the presence of active transcription factors. This model consists of nine reactions where one reaction is not described by mass-action kinetics, which would make this system already hard to simulate for some software packages.

We modeled cell division in both an explicit and implicit manner (see Information S1 Section 5). Modeling cell division explicitly can be seen as a complicated time event where timing and assignments depend on distinct distributions. We consider gamma distributed event waiting times for cell division. At a cell division event the molecular content of the mother cell is binomially partitioned over its two daughter cells. This is in contrast with earlier work from Kierzek et al. [25] who used a fixed generation time and divided the molecular content into two. Commonly used model definition formats (e.g. as encodable in SBML) do not support such events, which makes a sequential simulation approach a necessity. In such a scenario, each simulation starts with the results of the previous simulation. For these reasons, modeling cell division explicitly is inconvenient and non-trivial, especially for graphical user interface (GUI) based simulators. In the implicit cell division model growth rate is incorporated as a first-order rate constant that continuously dilutes cellular components into new cells.

**Figure 7:**
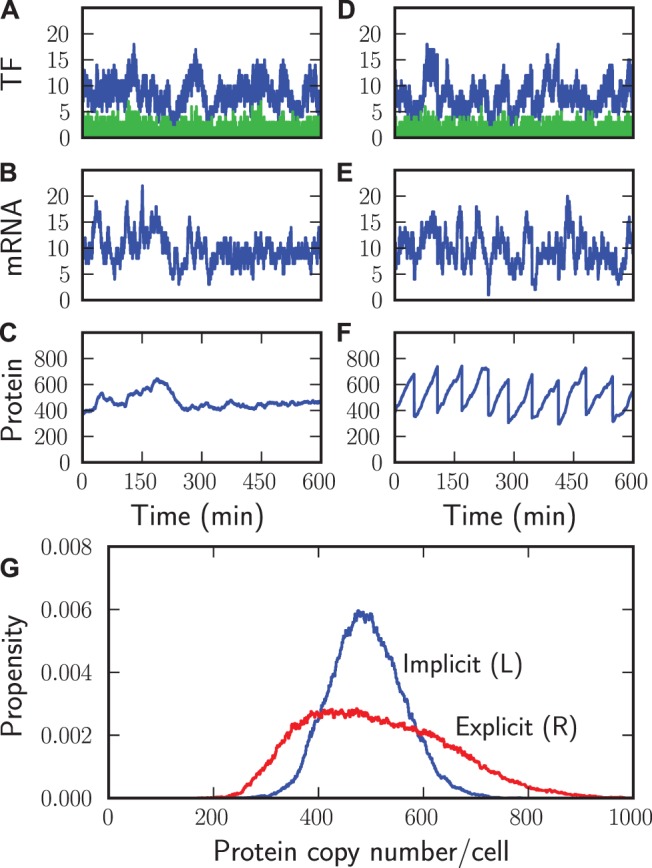
Modeling single-cell transcription and translation with and without cell division. StochPy plots of simulating stochastic gene expression. Modeling details of cell division periods: Gamma-distributed with scale parameter is 60.0 and shape parameter is 1.0. Implicit and explicit time series of transcription factor copy numbers (A and D), mRNA copy numbers (B and E), and protein copy numbers (C and F). Distributions of protein copy numbers for modeling cell division explicitly and implicitly (G). The model is further described in Information S1 Section 5.

The differences between modeling cell division explicitly and implicitly are illustrated in Figure 7. Without incorporating cell division explicitly the dynamics of transcription factors, mRNA, and proteins are different (Figures 7A–F). Most apparent are the differences for the protein copy numbers, which quickly reached a steady state (Figure 7C). In contrast, explicit modeling of cell division caused large variations in the protein copy numbers (Figure 7F). As a result, the distribution of mean protein copy numbers was significantly different if cell division was explicitly incorporated (Figure 7G). These differences are also observable for transcription factors (active, inactive) and mRNA (not shown).

### Benchmarking StochPy

The StochPy software features and speed performance were benchmarked against widely used, existing stochastic software to make a fair and broad comparison of available tools for stochastic simulations.

#### Feature comparison with existing stochastic tools

In an extensive search for available stochastic simulators, CAIN, COPASI, Facile-EasyStoch, GillespieSSA, and StochKit2 were identified as those with the closest functionality to StochPy. Here, we will discuss and compare these tools against StochPy through a feature comparison. A comprehensive feature comparison is provided in Table 1.

**Table 1:** Feature comparison between StochPy and existing (stochastic) software.

Feature	CAIN	COPASI	EasyStoch	GillespieSSA	StochKit2	StochPy
**Implemented solvers:**						
- Exact SSA	•	•	•	•	•	•
- Inexact SSA	•	•		•	•	•
**Simulator options:**						
- SBML support	○^1^	•			○^2^	•
- Human interpretable input			•			•
- Stochastic test suite						•
- Extrinsic noise			•			
- Explicit output			•	•	○^3^	•
- Fixed-interval output	•	•	•		•	•
**Output analysis:**						
- Auto-correlations						•
- Histogram distance	•				•	
- Propensities		•				•
- Moments						•
- Waiting times						•
**Software characteristics:**						
- Plotting facilities	•	•		•	○^4^	•
- Data exportation	•	•	•		•	•
- GUI	•	•				
- Flexible environment				•		•

Summary of features offered in StochPy and other stochastic modeling software.

•: Feature is present.

○ : Feature is partially present or requires additional dependencies.

Notes: 1. Limited ability to parse kinetic laws: Complicated expressions may not parsed. 2. Not all SBML documents can be converted into the StochKit2 model format. 3. Provided as an add-on functionality of StochKit2, whereas with limited options compared to the default installation of StochKit2. 4. Only if proprietary software (MATLAB) is installed.

1. *Explicit stochastic simulation output.* An important distinction between StochPy and most other stochastic simulators is in their output: Explicit rather than fixed-interval output is used and has several implications. Firstly, fixed-interval output does not allow the calculation of event waiting times, a unique feature provided by StochPy. Secondly, handling of fixed-interval output requires expert knowledge of the modeler (how many fixed intervals are sufficient to do a certain type of analysis properly). Thirdly, determining the minimal number of fixed intervals necessary is time-consuming. In the StochPy software one can exploit the convergence of higher moments to determine the minimal number of time steps necessary to accurately determine certain model properties. However, the benefit of using fixed-interval output is that less data can be stored which gives this approach a significant speed advantage. This can be useful if, for instance, only moments and time series are of interest and not probability distributions.
2. *SBML support and simulating diverse stochastic models.* Neither Facile-EasyStoch nor GillespieSSA provide SBML support while CAIN and StochKit2 support only a subset of available SBML models. As a consequence, many models (e.g. those with time and particle-number events or complicated rate laws) cannot be simulated, which limits their general purpose simulation capabilities. In contrast, both COPASI and StochPy support SBML levels 1–2 (V4). However, COPASI does not support (SBML) events in stochastic simulations and as a result, in our comparison StochPy is the only simulator able to successfully pass all tests in the SBML stochastic test suite [22].
3. *Analysis of stochastic data.* Besides various (unique) numerical analysis techniques StochPy also provides preprogrammed plotting functions. Calculation of event waiting times, propensity probability distributions, (co-)variances, and auto-correlations for one or more generated trajectories are currently unique numerical analysis techniques of StochPy. In addition, analysis of time series of species and propensities, probability distributions of species amounts, and moments can be done within StochPy. While not shown in the case studies, StochPy can calculate and visualize the average of multiple time trajectories for time series, auto-correlations, and distributions.
4. *Flexible environment for interactive modeling.* Due to its flexible design StochPy’s functionality can be extended far beyond saving data files and preprogrammed plotting capabilities. By integrating its functionality with industrial strength Python scientific libraries (e.g. Matplotlib [18], NumPy [26] and SciPy [27]) sophisticated user-defined analysis methods can be seamlessly applied to the output of StochPy simulations.

#### Direct solver performance

The direct solver of StochPy was benchmarked against the direct solvers of two widely used and high-performance stochastic tools i.e. CAIN and StochKit2 (both implemented in C++) for various stochastic models, the results of which are shown in Table 2. We have specifically chosen CAIN and StochKit2, because those are the fastest solvers currently available.

**Table 2:** Speed performance benchmark between StochPy and existing (stochastic) software.

Simulation Type	CAIN	CAIN (API)	StochKit2	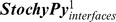
Small	0.7–0.10	0.24–0.10	0.24–0.07	1.0–0.31
*Non mass-action*	0.5–0.10	N/A	96–0.16	1.0–0.45
*Parallel*	0.04–0.07	0.04–0.06	0.03–0.18	0.28–0.33
*Parallel & time events*	1.9–1.9^2^	N/A	1.7–0.18	1.0–0.3
*Parallel & particle number events*	3.2–3.7^2^	N/A	1.5–0.18	1.0–0.3
*Assignments*	N/A	N/A	N/A	1.0–1.0
Large	0.14	0.28	0.09	0.56
XL	0.15	0.31	0.11	0.66
XXL	0.24	0.37	0.11	0.93

Results of benchmarking the direct method of StochPy. Simulation time was divided by the simulation time of the StochPy solvers: StochPy’s solver was faster if the reported ratio’s are larger than one and vice versa. A “−” indicates that short and long simulations were done to illustrate the potential difference between them. N/A is shown if the simulator was not possible to perform the simulation. For parallel simulations, 100 trajectories were done. In each comparison the number of fixed intervals was equal to the number of time steps in the simulation. Simulations were done on a Intel Core i5-2430M CPU 2.40 GHz×4 64-bit with Ubuntu 12.04 LTS as operating system. Stochastic models and a script to simulate these models within StochPy are available in Scripts S2.

Notes:

1 StochPy with interfaces to CAIN and StochKit2. Simulation time includes time to parse results into StochPy.

2 Cain cannot parse events, so the user most specify them in the GUI.

3 Optimal theoretical result without including time to merge the output of all sequential simulations.

To fairly compare StochPy’s performance against these other tools, the number of fixed-intervals was set equal to number of time steps in the stochastic simulation. Note that determining the minimal number of fixed-intervals necessary to perform a particular analysis requires doing multiple simulations (as shown in Figure 2), which makes this fixed-interval approach less efficient and more time-consuming than Table 2 reports.

The first conclusion from this benchmark is that no single solver was the fastest in any case. Secondly, StochPy is the only stochastic simulator that was able to correctly simulate all stochastic models tested in this benchmark. This in contrast to the CAIN API which can accept models consisting of only mass-action kinetics.

Thirdly, for different numbers of simulation time steps, significant differences in simulation time were found for different solvers. For relatively short simulations, StochKit2’s performance is reduced, as it requires substantial time to compile models with e.g. events and non mass-action kinetics. This effect is negligible for relatively long simulations. Both the CAIN and StochKit2 solvers outperformed StochPy’s direct solver for most tested models if simulations were done for a relatively large number of time steps (except e.g. modeling events with CAIN).

Fourthly, StochPy’s performance increased with respect to the performance of both CAIN and StochKit2 when larger modes were considered. For instance, CAIN needed about 4 minutes to parse the largest model tested (parsing time was omitted from the benchmark), while both StochKit2 and StochPy were able to parse this model within seconds. For relatively long simulations of models with many species, StochKit2’s solvers were about 10 times faster than those of StochPy, which is expected because our software is written in Python rather than C++.

Since, we also offer access to CAIN and StochKit2 solvers directly from StochPy, we also tested the speeds of CAIN and StochKit2 for this mode of operation. While exploiting these solvers in StochPy appears slower than the native application, this time only includes parsing of the simulation output for post-simulation analysis. This can take a significant amount of time for large data sets.

**Figure 8:**
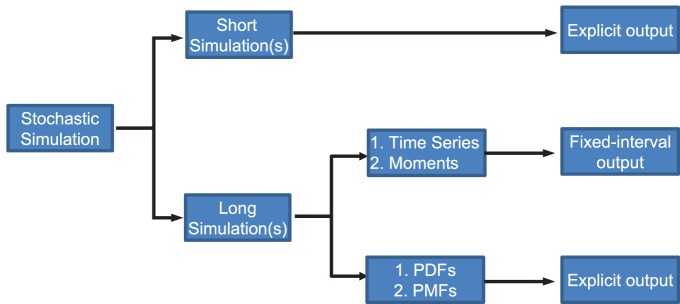
Stochastic modeling Decision Tree. Both fixed-interval and explicit output have their advantages and disadvantages. The decision whether to use fixed-interval or explicit output depends on the type of analysis.

As StochPy provides access to multiple SSAs, SSA implementations, and simulation tools and as discussed above, there is no ‘one size fits all’ approach, we provide a decision tree to help guide prospective modelers in how best to select a method that suits their model (see Figure 8). Here, decisions are made depending on the simulation time and the output of the solver. Insights into time series or moments can be easily obtained with solvers that provide fixed-interval output, whereas solvers that provide explicit output are, in principle, better suited for determining probability distributions of molecule copy numbers and event waiting times.

## Conclusions

Stochastic modeling in systems biology demands a certain level of flexibility in simulation, management of stochastic models and the handling of simulation data. Depending on the size of the system of interest and its degrees of time-scale separation, the different SSAs each have their particular (dis-)advantages. The differences in simulation time between stochastic simulation packages are often due to the fixed-interval reporting of simulation data versus the use of explicit output. To achieve the accuracy of explicit solvers the differences in simulation time greatly reduce, and ultimately boil down to, differences in the programming languages. In systems biology applications, often the pure simulation data rather than the fixed-interval simulation data is of interest. The pure simulation data allows for the accurate determination of various time and copy number associated probability measures.

We presented StochPy as a versatile modeling package for stochastic simulation of molecular control networks inside living cells that provides solvers which return explicit stochastic simulation output. Its integration with Python’s scientific libraries and PySCeS makes it an easily extendible and a user-friendly stochastic simulator package. We highlighted this by implementing both the solvers of CAIN and StochKit2 that return only fixed-interval output, which can be useful for obtaining insight into time series and moments. The high-level statistical and plotting functions of StochPy allow for quick and interactive model interrogation at the command-line. Python’s scripting capabilities allow for more complicated and in-depth analysis of stochastic models and meets many of the demands for systems biology.

## Supporting Information

Dataset S1 **The results of testing StochPy against the SBML stochastic test suite.**
(ZIP)

Scripts S1 **Annotated scripts and input files used to generate modeling results.**
(ZIP)

Scripts S2 **Stochastic models and a script to simulate the models used for benchmarking StochPy.**
(ZIP)

Information S1 **Additional information about the different models that were used as examples to demonstrate the capabilities of StochPy.**
(PDF)
