# Supplementary material for: StochPy: A Comprehensive, User-Friendly Tool for Simulating Stochastic Biological Processes
Source: PLoS One. 2013 Nov 18;8(11):e79345. doi: 10.1371/journal.pone.0079345 (PMC3832486; doi:10.1371/journal.pone.0079345)
Supplement: Information S1 — Additional information about the different models that were used as examples to demonstrate the capabilities of StochPy. (PDF) [file pone.0079345.s004.pdf]

StochPy - Supplementary Information S1

Timo R. Maarleveld, Brett G. Olivier, Frank J. Bruggeman

This file contains, besides an explanation of analyzing stochastic simulation output, additional information about the different models that were used as examples to demonstrate the capabilities of StochPy. For convenience in each model the cell volume was set to 1 (cell volume of E. coli is about 1 fl). Within StochPy simulations were done with $2.0 \cdot 10^6$ time steps unless stated otherwise.

1 Analyzing stochastic simulation output

The stochastic simulation output is not discretized and, as a consequence, StochPy cannot calculate the mean of a particular species by taking the average over the vector of species quantities. First, the species state probability, the probability of species X in a given state n, has to be calculated by determining the fraction of time spent in this particular state,

$$P(X = n) = \frac{\sum_{j=1}^{M} (T_j | X = n)}{T_{sim}} \quad (1)$$

Here, T_j is a time period that species X spent in state n, and T_{sim} is the total simulation time. For the species X_1 shown in Figure 1 of the manuscript, as first sight, one might think that $P(X_1 = 0) << P(X_1 = 1)$ because $X_1 = 1$ during 19 of the 20 simulated time steps. In stochastic simulations, reaction events are irregular due to their stochastic nature. In this particular example, the first reaction event took 39.976 time units, while the next 19 reaction events together took only 1.002 time units. Therefore, the probabilities of being in state zero and one are $P(X_1 = 0) = 39.976/40.978 \approx 0.975$ and $P(X_1 = 1) = 1.002/40.978 \approx 0.025$ and therefore $P(X_1 = 0) >> P(X_1 = 1)$.

Subsequently, StochPy can determine the mean of this species by taking the sum of the product of species state probabilities and species state values,

$$\langle X \rangle = \sum_{i=1}^{N} P(X = n_i) \cdot n_i \quad (2)$$

Since we already calculated the species state probabilities of species X_1, we can simply determine its mean for the done simulation, $\langle X_1 \rangle = P(X_1 = 0) \cdot 0 + P(X_1 = 1) \cdot 1 \approx 0.025$. Both Equations (1) and (2) can also be exploited for calculating propensity probabilities and means. Then, a_{R_i} is the propensity of reaction R_i and $P(a_{R_i} = n)$ is the propensity probability of reaction R_i for state n. For instance, $P(a_{R_1} = 0) = 39.976/40.978 \approx 0.975$ and $P(a_{R_1} = 0.05) \approx 0.025$ and therefore $\langle a_{R_1} \rangle = P(a_{R_1} = 0) \cdot 0 + P(a_{R_1} = 0.05) \cdot 0.05 \approx 1.25 \cdot 10^{-3}$.

Returning explicit output allows StochPy, besides e.g. discrete plotting, to provide the unique feature of the calculation and analysis of event waiting times. The event waiting times for a particular reaction is a vector of inter-event times of that particular reaction. For a given reaction R_i these waiting times can be calculated by determining all its inter-event times,

$$\mathbf{w}(R_i) = \forall\, j : (t_{j+1} | R_i) - (t_j | R_i) \quad (3)$$

Here, $\mathbf{w}(R_i)$ are the event waiting times of reaction R_i and t_j is an event time.

Moreover, StochPy provides the unique feature of auto-covariance and auto-correlation analysis of species and propensities.

Covariance is a measure of two random variables of their joint variability. Therefore, auto-covariance is the covariance of a variable against a time-shifted version of itself. We can subsequently determine the

auto-correlation by dividing the auto-covariance by the variance of the signal. For this reason, (auto-)correlation is a dimensionless property which makes it often a more useful measure of correlation than (auto-)covariance. In StochPy, before calculating both auto-covariances and auto-correlations the explicit output (of which an example is shown in Figure 1 of the manuscript) is transferred to a regular grid. Without a regular grid, the number of different lags could be to $0.5N \cdot (N-1)$ where N is the number of time points.

Next, both the auto-covariance and the auto-correlation can be calculated by determining the signal difference for a given time lag τ. We illustrate this for species X_i, but the same method can be used for propensity a_{R_i}. First, we determine the auto-covariance of species X_i for a given time lag τ,

$$ACOV(\tau) = \frac{1}{N} \cdot \sum_{t=1}^{N-\tau} (X_i(t) - \langle X_i \rangle)(X_i(t+\tau) - \langle X_i \rangle) \tag{4}$$

where $X_i(t)$ is the species state at time t. Accordingly, the auto-correlation of X_i for a given time lag τ can be determined by dividing $ACOV(\tau)$ with the signal variance,

$$ACOR(\tau) = \frac{ACOV(\tau)}{\frac{1}{N} \cdot \sum_{t=1}^{N} (X_i(t) - \langle X_i \rangle)^2} \tag{5}$$

2 Example 1: Modeling single-cell transcription

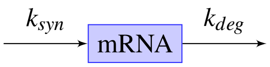

Figure 1. Immigration-death model. k_{syn} and k_{deg} denote the mRNA synthesis and degradation rate constant, respectively.

The network shown in Figure 1 consists of two reactions: The synthesis and degradation of mRNA occurs with a zero-order reaction equal to k_{syn} and with a first-order reaction equal to $k_{syn}\cdot$ mRNA, respectively. The mRNA synthesis rate, k_{syn}, was 10 min^{-1} during our simulations. The mRNA degradation rate varied during our simulations between $0.01 - 0.5$ min^{-1}.

In steady state the synthesis rate equals the degradation rate, $k_{syn} - k_{deg} \cdot \langle mRNA \rangle = 0$, and therefore,

$$\langle mRNA \rangle = \frac{k_{syn}}{k_{deg}} \tag{6}$$

Hence, the mean mRNA copy number per cell depends only on the used parameters values. For $k_{syn} = 10$ min^{-1} and $k_{deg} = 0.2$ min^{-1} the analytical $\langle mRNA \rangle$ is 50. Using StochPy's direct solver, we determined both the mean and the variance of mRNA copy numbers per cell at about 50.

This immigration-death model is one of the simplest examples of a Poisson process. A Poisson process is a stochastic process where events occur continuously and independently of one another. Therefore, the mean mRNA copy number is identical to its variance. For a Poisson distribution the probability mass function of mRNA is given by,

$$f(n, \lambda) = P(mRNA = n) = \frac{\lambda^n \cdot e^{-\lambda}}{n!} \tag{7}$$

where λ is the mean mRNA copy number and its variance and n is the mRNA copy number. This probability distribution is shown in the manuscript in Figure 1C.

The story is a bit different for propensities. The propensity a_{R_1} is constant, while the propensity of a_{R_2} depends on parameter k_2 and the mRNA copy number. We already determined that the mRNA copy number is Poisson distributed. By multiplying the mean and variance with a constant (k) and the squared of this constant, we can calulate the mean and variance of a_{R_2},

$$\begin{aligned} E(ax) &= k \cdot E(x) \\ Var(ax) &= k^2 \cdot Var(x) \end{aligned} \tag{8}$$

Hence, the mean and variance of a_{R_2} are 10 and 2, respectively. The simulation results of StochPy were in agreement with these analytical results. Within StochPy, we can also determine the probability of a certain propensity, which is analytically given by,

$$P(a_{R_2} = y) = P(mRNA \cdot k_{deg} = y) = P(mRNA = \frac{y}{k_{deg}}) \tag{9}$$

Furthermore, we used the "change of variable" method to determine the analytical solution of the probability mass function of a_{R_2},

$$f(y, \lambda) = P(a_{R_2} = y) = \frac{\lambda^{y/k_{deg}} \cdot e^{-\lambda}}{(y/k_{deg})!} \qquad \forall \ \frac{y}{k_{deg}} \in n \tag{10}$$

and otherwise 0. This is, in fact, a Poisson distribution whose x-axis is distorted.

For this model the auto-correlation function is also analytically known [1],

$$ACOR(\tau) = e^{-k_{deg} \cdot \tau} \tag{11}$$

Thus, the auto-correlation of mRNA copy numbers does not depend on its synthesis rate. For different k_{deg} values the StochPy simulations done until t = 20.000 were in agreement with the analytical solution given in Equation (11), which is shown in Figure 3E of the manuscript.

3 Example 2: Modeling bursty single-cell transcription

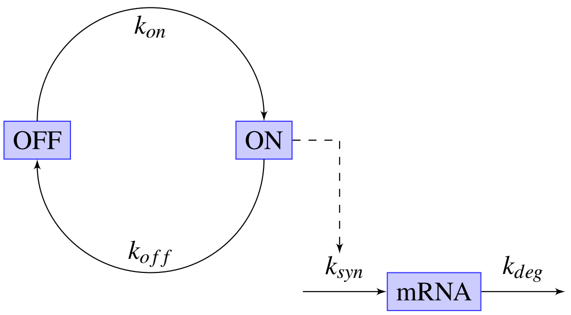

Figure 2. Network that describes single-cell transcription in a stochastic manner. k_{on}, k_{off}, k_{syn}, and k_{deg} denote the ON switching, OFF switching, synthesis, and degradation rate constant, respectively.

Figure 2 illustrates a network that consists of a switching gene and mRNA turnover. Product mRNA is synthesized only in the ON state. This network is composed out of the following reactions,

$$\begin{aligned} OFF &\xrightarrow{k_{on} \cdot OFF} ON \\ ON &\xrightarrow{k_{off} \cdot ON} OFF \\ &\xrightarrow{k_{syn} \cdot ON} mRNA \xrightarrow{k_{deg} \cdot mRNA} \end{aligned} \tag{12}$$

The mRNA synthesis and degradation rate constants were 80 min^{-1} and 2.5 min^{-1} in our simulations, respectively. Switching from ON to OFF state and vice versa occurred at a rate of 0.05 min^{-1} during bursty transcription and at a rate of 5.0 min^{-1} during non-bursty transcription, respectively. Therefore, lifetimes of the ON and OFF state ($1/k_{off}$ and $1/k_{on}$) were hundred times larger during bursty transcription than during non-bursty transcription.

The mean mRNA copy number per cell and the fraction of time spent in the ON and OFF state are as follows,

$$\begin{aligned} \langle mRNA \rangle &= \frac{k_{syn}}{k_{deg}} \cdot \frac{k_{on}}{k_{on} + k_{off}} \\ \langle ON \rangle &= \frac{k_{on}}{k_{on} + k_{off}} \\ \langle OFF \rangle &= \frac{k_{off}}{k_{on} + k_{off}} \end{aligned} \tag{13}$$

Because both switching probabilities are equal, the fraction of time spent in both states should be equal. Therefore, the mean mRNA copy number per cell during bursty and non-bursty transcription is equal. Based on our parameters the mean mRNA copy number per cell should be equal to 16. The standard deviation of the mRNA copy number, however, differs between bursty and non-bursty transcription, which can be calculated as follows,

$$\sigma_X = \sqrt{\langle X^2 \rangle - \langle X \rangle^2} \tag{14}$$

StochPy simulations gave that $\langle mRNA \rangle$ was about 16.0 copy numbers per cell for bursty and non-bursty transcription, respectively. The fraction of time spent in the ON and OFF state — $\langle OFF \rangle$ and $\langle ON \rangle$ — for both bursty and non-bursty transcription was about 0.50. For a single StochPy simulation of 10^6 steps, standard deviations were $\sigma_{mRNA} \approx 16.20$ and $\sigma_{off} = \sigma_{on} \approx 0.50$ for bursty transcription and $\sigma_{mRNA} \approx 8.21$ and $\sigma_{off} = \sigma_{on} \approx 0.50$ for non-bursty transcription.

With StochPy we also determined the probability distribution of mRNA copy numbers. The exact probability of having m mRNA copy numbers [3] at steady state is given by,

$$P_m(m) = \frac{m_s^m e^{-m_s}}{m!} \frac{\Gamma(\zeta_0 + m)\Gamma(\zeta_0 + \zeta_1)}{\Gamma(\zeta_0 + \zeta_1 + m) + \Gamma(\zeta_0)} 1F_1(\zeta_1, \zeta_0 + \zeta_1 + m; m_s) \tag{15}$$

where Γ denotes the gamma function, $m_s = k_{syn}/k_{deg}$, $\zeta_0 = k_{on}/k_{off}$, $\zeta_1 = k_{off}/k_{deg}$, and $1F_1(a, b; z)$ is the Kummer confluent hyper-geometric function of the first kind. The behavior of the exact mRNA distribution can be bimodal and unimodal as is illustrated in the manuscript in Figure 4A.

For this model the waiting time probability density function [2] is given by,

$$\begin{aligned} f(t) &= P[X \in (t, t + dt)]/dt = w_1 r_1 e^{-r_1 t} + w_2 r_2 \\ r_{1,2} &= (K \pm \sqrt{K^2 - 4 k_{syn} k_{on}}), r_1 > r_2 \end{aligned} \tag{16}$$

where $w_1 = 1 - w_2 = (k_{syn} - r_2)/(r_1 - r_2) \in (0, 1)$ and $K = k_{off} + k_{on} + k_{syn}$. In addition, the analytical mean waiting times were compared with those obtained from the stochastic simulation,

$$\langle t_{mRNA} \rangle = \int_0^\infty t f(t) dt = \frac{w_1}{r_1} + \frac{w_2}{r_2} = \tau_{syn} \left(\frac{\tau_{on}}{\tau_{on} + \tau_{off}} \right)^{-1} \tag{17}$$

The waiting times of mRNA for bursty and non-bursty transcription derived with StochPy are equal to the analytical waiting times of mRNA in both transcription modes ($\langle t_{mRNA} \rangle = 0.25$). Finally, the noise in the mRNA copy number is equal to,

$$\frac{\sigma^2_{mRNA}}{\langle mRNA \rangle^2} = \frac{1}{\langle mRNA \rangle} + \frac{\tau_o}{\tau_o + \tau_{mRNA}} \cdot \frac{\sigma^2_{ON}}{\langle ON \rangle^2} \tag{18}$$

where $\tau_o = 1/(k_{off} + k_{on})$, $\tau_{mRNA} = 1/(k_{deg})$, and $\sigma^2_{ON}/\langle ON \rangle^2 = k_{off}/k_{on}$. The analytical mRNA noise in bursty and non-bursty transcription is 1.02 and 0.26, respectively. A single StochPy simulation of 10^6 steps gave 1.03 and 0.26 for bursty and non-bursty transcription, respectively.

4 Example 3: Modeling single-molecule Michaelis-Menten kinetics

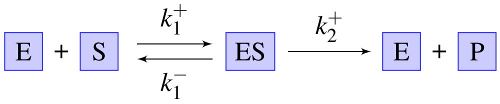

Figure 3. The classic enzyme kinetic scheme. k_1^+,k_1^-, and k_2^+ denote rate constants.

Michaelis-Menten kinetics have been used already for one-hundred years for modeling enzyme kinetics for a large ensemble of enzyme molecules. The simplest enzyme mechanism considers an enzyme, E, which converts a single substrate S into a single product P by first forming an enzyme-substrate complex ES. An illustration of this system is shown in Figure 3. Recent advances in single-molecule measurements allow the study of enzymatic reactions at the level of single enzymes. Modeling of single-molecule enzymology requires stochastic simulations. By performing single-molecule experiments the probability density, $f_T(t)$, of event waiting times T for product P arrivals can be determined [4, 5].

Here, we stochastically modeled single-molecule enzyme kinetics to demonstrate the capabilities of StochPy. Single-molecule enzyme kinetics means that, at all times, the total enzyme copy number is 1,

$$E[t] + ES[t] = 1 \tag{19}$$

Because the substrate depletion with one enzyme copy number is negligible, the substrate S copy number can be considered constant,

$$S[t] = S = c \tag{20}$$

Subsequently, we can write the rate equations of this system in terms of time dependent probabilities [4],

$$\begin{aligned} \dot{P}_E(t) &= -k_1^+ \cdot P_E(t) + k_1^- \cdot P_{ES}(t) \\ \dot{P}_{ES}(t) &= k_1^+ \cdot P_E(t) - (k_1^- + k_2^+) \cdot P_{ES}(t) \\ \dot{P}_P(t) &= k_2^+ \cdot P_{ES}(t) \end{aligned} \tag{21}$$

The probability for a single enzyme to be in the unbounded state is the fraction of time spent the unbounded state. Similarly, the probability for a single enzyme to be in the bounded state is the fraction of time spent in the bounded state. We can write these probabilities in terms of rate constants and substrate copy numbers,

$$\begin{aligned} P_E &= \frac{\tau_E}{\tau_E + \tau_{ES}} = \frac{k_1^- + k_2^+}{k_1^- + k_2^+ + k_1^+ \cdot S} \\ P_{ES} &= \frac{\tau_{ES}}{\tau_E + \tau_{ES}} = \frac{k_1^+ \cdot S}{k_1^- + k_2^+ + k_1^+ \cdot S} \end{aligned} \tag{22}$$

These probabilities equal the analytical $\langle E \rangle$ and $\langle ES \rangle$ copy numbers in steady state. StochPy simulations gave that $\langle E \rangle \approx 0.555$ and $\langle ES \rangle \approx 0.445$ which is in agreement with analytical values for the parameter values used ($S \cdot k_1^+ = 2/3$, $k_1^- = 1/12$, and $k_2^+ = 3/4$).

Next, the steady-state flux of this system is given by,

$$V_{ss} = k_1^+ \cdot S \cdot P_E - k_1^- \cdot P_{ES} = k_2^+ \cdot P_{ES} \tag{23}$$

From this relationship we can derive the famous Michaelis-Menten relationship,

$$\begin{aligned} V_{ss} = k_2^+ \cdot P_{ES} &= \frac{k_2^+ \cdot k_1^+ \cdot S}{k_1^+ \cdot S + k_1^- + k_2^+} \\ &= \frac{k_2^+ \cdot S}{S + \frac{k_1^- + k_2^+}{k_1^+}} \\ &= \frac{V_{max} \cdot S}{S + K_m} \end{aligned} \tag{24}$$

Here, $V_{max} = k_2^+$ and $K_m = \frac{k_1^- + k_2^+}{k_1^+}$. Then, for large ensembles the product P copy number at time t depends on,

$$P(t) = V_{ss} \cdot t \tag{25}$$

For single-molecule enzymology experiments the product P copy number fluctuates around this analytical solution for large ensembles. In Figure 6C of the manuscript three Monte Carlo trajectories that describe the time evolution of this model are shown to illustrate these fluctuations for small ensembles.

Next, the probability density of event waiting times for product P arrivals is given by,

$$f_T(t) = k_2^+ \cdot P_{ES}(t) \tag{26}$$

The rate equations given in Equation (21) represent a system of linear first-order differential equations which can be solved analytically for $P_E(t)$, $P_{ES}(t)$, and $P_P(t)$. Therefore, using Mathematica [6] we can analytically determine $f_T(t)$,

$$f_T(t) = k_2^+ \cdot \frac{e^{-0.5(k_1^+ \cdot S + k_1^- + k_2^+ + \sqrt{A}) \cdot t} \cdot (-1 + e^{\sqrt{A} \cdot t}) \cdot k_1^+ \cdot S}{\sqrt{A}} \tag{27}$$

where A depends on the rate constants and the substrate copy number S,

$$A = -4 \cdot k_1^+ + k_2^+ \cdot S + (k_1^+ \cdot S + k_1^- + k_2^+) \tag{28}$$

Rearrangement will show that this analytical result is identical to those obtained by Kou et al. [4]. For single-molecule enzymology $f_T(t)$ peaks as is shown in the manuscript in Figure 6D.

It can be shown that the first raw moment of $f_T(t)$—the mean waiting time $\langle T \rangle$ of product P arrivals—is the inverse of the classic Michaelis-Menten equation derived in Equation (24),

$$\langle T \rangle = \frac{k_1^- + k_2^+}{k_1^+ \cdot k_2^+ \cdot S} + \frac{1}{k_2^+} = \frac{k_1^+ \cdot S + k_1^- + k_2^+}{k_2^+ \cdot k_1^+ \cdot S} = \frac{1}{V_{ss}} \tag{29}$$

Theoretically, $\langle T \rangle = 3.0$ and $V_{ss} = 1/3$ given the set of used parameters. StochPy simulations gave that $\langle T \rangle$ was about 3.0.

5 Example 4: Modeling regulated gene expression dynamics

Figure 4 illustrates the network that was used in this paper to demonstrate the flexibility of StochPy. This network consists, besides mRNA and protein, of active and inactive transcription factors. These

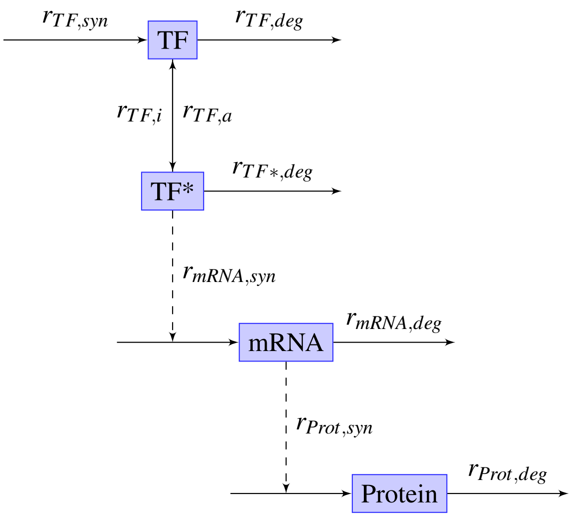

Figure 4. Network that describes regulated gene expression dynamics in a stochastic manner. $r_{X,syn}$, and $r_{X,deg}$ denote the synthesis and degradation rate constant for X (mRNA, TF, and protein), respectively.

transcription factors switch between an inactive and an active state where the active state stimulates mRNA synthesis. Protein synthesis can occur if mRNA is present in the cell.

This network is composed out of the following reactions,

$$\begin{aligned}
&\xrightarrow{k_{tf,syn}} TF \xrightarrow{k_{tf,deg}\,\cdot\,TF} \\
&TF \xrightarrow{k_{tf,a}\,\cdot\,TF} TF* \xrightarrow{k_{tf,deg}\,\cdot\,TF*} \\
&TF* \xrightarrow{k_{tf,i}\,\cdot\,TF*} TF \\
&\xrightarrow{\delta} mRNA \xrightarrow{k_{m,deg}\,\cdot\,mRNA} \\
&\xrightarrow{k_{p,syn}\,\cdot\,mRNA} Protein \xrightarrow{k_{p,deg}\,\cdot\,Protein}
\end{aligned} \tag{30}$$

where δ is $(k_{m,syn} \cdot TF*)/(TF* + k_X)$ with $k_X = 2$. The transcription factor synthesis and degradation rates were 0.61 min^{-1} and 0.05 min^{-1}. Switching from active to an inactive transcription factor and vice versa occurred at a rate of 5 min^{-1} and 0.5 min^{-1}, respectively. The mRNA synthesis and degradation rates were 1.36 min^{-1} and 0.10 min^{-1}, while the protein synthesis and degradation rates were 1.13 min^{-1} and 0.011 min^{-1} in our simulations.

Each living cell grows, doubles its content, and subsequently divides which has an effect on gene expression dynamics. Cell division can be modeled explicitly and implicitly. Explicit modeling of cell division means that the cell content is binomially distributed between two daughter cells after a certain generation time T_g. This generation time is gamma distributed, but on average the cellular content is doubled after T_g. Note that population averages also depend on the age distribution in the population. We assume a constant age distribution (a synchronous population). This allows us to track only one daughter cell after each cell division rather than tracking the complete phylogenetic tree.

Alternatively, in implicit modeling the effect of protein dilution due to growth and division is described

by including the growth rate (μ) into the degradation rates of transcription factors, mRNA, and protein,

$$
\begin{aligned}
&\xrightarrow{k_{tf,syn}} TF \xrightarrow{(k_{tf,deg}+\mu)\cdot TF} \\
&TF \xrightarrow{k_{tf,a}\cdot TF} TF* \xrightarrow{(k_{tf,deg}+\mu)\cdot TF*} \\
&TF* \xrightarrow{k_{tf,i}\cdot TF*} TF \\
&\xrightarrow{\delta} mRNA \xrightarrow{(k_{m,deg}+\mu)\cdot mRNA} \\
&\xrightarrow{k_{p,syn}\cdot mRNA} Protein \xrightarrow{(k_{p,deg}+\mu)\cdot protein}
\end{aligned} \tag{31}
$$

We can determine the growth rate corresponding to a doubling time of 60 minutes voa the following relationship,

$$T = \frac{\ln(2)}{\mu} = 60\ min \tag{32}$$

Within StochPy, stochastic simulations were done for 5000 generations, about 10×10^6 time steps, to be able to accurately determine the mean copy numbers of transcription factors, mRNA, and protein (Table 1). Previous research showed that the average copy numbers with a constant age distribution were about 4% larger than with an exponential age distribution [7]. The results obtained with StochPy are in agreement with this finding. In addition, modeling cell division explicitly resulted in larger standard deviations for especially protein copy numbers.

Table 1. Analysis of explicit and implicit modeling of cell division

	**TF**		**TF***	
	μ	σ	μ	σ
Explicit modeling	1.04 ± 0.00	1.03 ± 0.00	9.26 ± 0.03	3.39 ± 0.01
Implicit modeling	1.01 ± 0.00	1.01 ± 0.00	9.01 ± 0.03	3.02 ± 0.01
	**mRNA**		**Protein**	
	μ	σ	μ	σ
Explicit modeling	9.99 ± 0.02	3.53 ± 0.01	508.13 ± 1.62	132.96 ± 0.59
Implicit modeling	9.79 ± 0.02	3.19 ± 0.01	489.35 ± 0.88	70.18 ± 0.47

Mean (μ) and standard deviation (σ) of TF, TF*, mRNA, and protein. For both μ and σ, the variation (1 SD) was based on 10 simulations.

References

1. Gardiner, C. W. (1997). Handbook of Stochastic Methods.

2. Dobrzynski, M. and Bruggeman, F. J. (2009). Elongation dynamics shape bursty transcription and translation. *PNAS*, **106**(8), 2583–2588.

3. Shahrezaei, V. and Swain, P. S. (2008). Analytical. distributions for stochastic gene expression. *PNAS*, **105**(45), 17256–17261.

4. Kou, S., Binny C., Wei M., Brian E., and Sunny X. (2005). Single-Molecule Michaelis-Menten Equations. *J. Phys. Chem.*, **109**, 19068–19081.

5. Qian, H. (2008). Cooperativity and Specificity in Enzyme Kinetics: A Single-Molecule Time-Based Perspective. *Biophysical Journal*, **95**, 10–17.

6. Wolfram, Stephen (1999) The MATHEMATICA® Book, Version 4, Cambridge university press.

7. Marathe, Rahul and Bierbaum, Veronika and Gomez, David and Klumpp, Stefan (2012). Deterministic and Stochastic Descriptions of Gene Expression Dynamics. *J Stat Phys*, 1–20.
